# PET imaging of amyloid with Florbetapir F 18 and PET imaging of dopamine degeneration with ^18^F-AV-133 (florbenazine) in patients with Alzheimer’s disease and Lewy body disorders

**DOI:** 10.1186/1471-2377-14-79

**Published:** 2014-04-09

**Authors:** Andrew Siderowf, Michael J Pontecorvo, Holly A Shill, Mark A Mintun, Anupa Arora, Abhinay D Joshi, Ming Lu, Charles H Adler, Douglas Galasko, Carolyn Liebsack, Daniel M Skovronsky, Marwan N Sabbagh

**Affiliations:** 1 Avid Radiopharmaceuticals, Philadelphia, PA, USA; 2 Banner Sun Health Research Institute, Sun City, AZ 10515W Santa Fe Dr, USA; 3 Mayo Clinic Arizona, Scottsdale, AZ, USA; 4 University of California, San Diego, CA, USA

**Keywords:** PET imaging, Alzheimer’s disease, Parkinson’s disease, Biomarkers

## Abstract

**Background:**

Biomarkers based on the underlying pathology of Alzheimer’s disease (AD) and Dementia with Lewy Bodies (DLB) have the potential to improve diagnosis and understanding of the substrate for cognitive impairment in these disorders. The objective of this study was to compare the patterns of amyloid and dopamine PET imaging in patients with AD, DLB and Parkinson’s disease (PD) using the amyloid imaging agent florbetapir F 18 and ^18^F-AV-133 (florbenazine), a marker for vesicular monamine type 2 transporters (VMAT2).

**Methods:**

Patients with DLB and AD, Parkinson’s disease (PD) and healthy controls (HC) were recruited for this study. On separate days, subjects received intravenous injections of florbetapir, and florbenazine. Amyloid burden and VMAT2 density were assessed quantitatively and by binary clinical interpretation. Imaging results for both tracers were compared across the four individual diagnostic groups and for combined groups based on underlying pathology (AD/DLB vs. PD/HC for amyloid burden and PD/DLB vs. AD/HC for VMAT binding) and correlated with measures of cognition and parkinsonism.

**Results:**

11 DLB, 10 AD, 5 PD, and 5 controls participated in the study. Amyloid binding was significantly higher in the combined AD/DLB patient group (n = 21) compared to the PD/HC groups (n = 10, mean SUVr: 1.42 vs. 1.07; p = 0.0006). VMAT2 density was significantly lower in the PD/DLB group (n = 16) compared to the AD/ HC group (n = 15; 1.83 vs. 2.97; p < 0.0001). Within the DLB group, there was a significant correlation between cognitive performance and striatal florbenazine binding (r = 0.73; p = 0.011).

**Conclusions:**

The results of this study show significant differences in both florbetapir and florbenazine imaging that are consistent with expected pathology. In addition, VMAT density correlated significantly with cognitive impairment in DLB patients (ClinicalTrials.gov identifier: NCT00857506, registered March 5, 2009).

## Background

Alzheimer’s disease (AD) is the most frequent cause of dementia in the US, and is responsible for up to 70% of cases [1]. Dementia with Lewy Bodies (DLB) is the second most common degenerative dementia, accounting for approximately 15%-30% of cases at autopsy [2]. Based on clinical features, diagnostic criteria have been developed to differentiate DLB from AD [2,3]. While these criteria improve classification, there is still considerable misdiagnosis compared to autopsy, particularly early in the disease course [4,5]. More accurate differentiation of these two conditions could provide clinically relevant prognostic information [6,7] and influence treatment decisions [8-10].

Biomarkers based on the underlying pathology of AD and DLB have the potential to improve diagnosis ^11^C-labeled Pittsburgh compound B (^11^C-PiB) was the first PET-ligand to selectively visualize β-amyloid in living patients [11]. The PET tracer florbetapir F 18 (Amyvid) differs from PiB as it has a longer half-life (109 min versus 20 min). Florbetapir PET correlates with beta-amyloid pathology at post-mortem [12]. PET and/or SPECT ligands that assess the integrity of the dopamine system have been available for several decades as indirect measures of dopaminergic neurodegeneration [13]. Florbenazine (AV-133) is a fluorinated VMAT-2 ligand for PET that has been shown to identify dopaminergic deficits in patients with PD and DLB [14,15].

The purpose of this study was to determine whether florbetapir PET would detect amyloid deposition in AD and DLB patients, and whether florbenazine PET would identify decreased VMAT2 density in PD and DLB patients. Secondary goals were to determine the reliability of expert interpretation in a cohort with a mixture of neuro-degenerative disorders and to further assess the safety and tolerability of florbetapir and florbenazine. This information would help determine whether the combination of florbetapir and florbenazine merit further investigation as a potential adjunct to clinical evaluation in the diagnosis of patients with dementia.

## Methods

### Study design

This study (AV133-B03 (ClinicalTrials.gov identifier: NCT00857506) was sponsored by Avid Radiopharmaceuticals (a subsidiary of Eli Lilly and Co.). The study was approved by the Institutional Review Boards (IRB) at the University of Pennsylvania, University of California at San Diego and Banner Sun Health Research Institute. Written informed consent was obtained from study participants and/or their authorized representatives prior to conduct of any study procedures. The study consisted of a screening visit and two imaging visits. Screening assessments included demographic information, safety assessment, including clinical laboratory assessment, vital signs and ECG, and an MRI scan. Each subject was imaged on 2 separate sessions not more than 4 weeks apart.

### Research participants

All subjects were >50 years of age and had to be able to tolerate two PET imaging sessions. AD and DLB subjects had to have a caregiver who could report on their mental status and activities of daily living. DLB subjects were enrolled if they met the diagnostic criteria for probable DLB [2] and did not meet the National Institute of Neurological and Communicative Disorders and Stroke and Alzheimer’s disease and Related Disorders Association (NINCDS-ADRDA) criteria for probable AD [16]. AD subjects were enrolled if they met the NINCDS criteria for probable AD, had a Mini Mental State Examination (MMSE) [17] score at screening between 10 and 24 inclusive and had no symptoms of parkinsonism. PD subjects were defined by research diagnostic criteria for probable PD [18] and did not have cognitive impairment based on the judgment of the enrolling investigator. HC subject were enrolled if they were cognitively normal and had no evidence of parkinsonism.

Patient groups were combined for some analyses. For analyses of florbetapir PET, the combined AD and DLB groups were compared to the combined PD and HC groups testing the hypothesis that AD and DLB patients would typically be amyloid positive (Aβ + PET scans) whereas PD and HC subjects would be, in general, amyloid negative (Aβ-). For analysis of florbenazine PET the combined PD and DLB groups were compared to the combined AD and HC groups.

### Clinical assessments

Demographic information including age, gender, education and disease duration (for patient groups) was collected for all subjects. Motor function was assessed using part III of the Unified Parkinson Disease Rating Scale (UPDRS) [19]. Motor examinations were performed in the practically defined “off” state, at least 12 hours after the last dose, in subjects receiving dopaminergic treatment [20]. Cognitive performance was rated with the MMSE. Non-serious adverse experiences were recorded for 24 hours after imaging and serious adverse experiences were collected for up to 30 days post-imaging.

### Scan acquisition

Subjects underwent a 10 minute acquisition (1×10 frame or 2×5 minutes frames), 50 minutes after intravenous injection of 10 mCi (370 MBq) of florbetapir F 18. For the florbenazine imaging session, an i.v. bolus of 5 mCi (185 MBq) of 18 F-AV-133 was administered. Brain scans were acquired over an approximately 10 min period, 50 minutes after injection. For both tracers, data was acquired from 3 sites, including 2 PET/CT scanners: Biograph 2 slice PET/CT and Biograph mCT 40 slice PET/CT and a dedicated PET scanner (ECAT HR+) all manufactured by Siemens Medical Solutions, Knoxville, TN. Images were reconstructed using iterative reconstruction. No post-reconstruction technique, such as smoothing or partial volume correction, was applied to the images.

### Image analysis

Five experienced nuclear medicine physicians independently reviewed all PET images. Readers had been trained to read both scans in a standardized fashion, and were blinded to clinical data. The interpretation given by the majority of the five readers’ for each image was used for analysis. Florbetapir scans were interpreted qualitatively and quantitatively as described previously [12,21,22]. For florbenazine, the readers conducted a binary assessment identifying the presence or absence of dopaminergic degeneration based on the intensity and distribution of florbenazine uptake in the striatum. Florbenazine images were spatially normalized to a standard atlas using a AV-133 PET template. Atlas-based volumes of interest (VOIs) were applied for target areas (caudate, anterior and posterior putamen and total striatum) and for the occipital cortex reference region. The ratio of tracer activity in target VOIs relative to the occipital cortex as a reference region was calculated to create standard uptake value ratios (SUVr).

### Statistical methods

The differences among diagnostic groups in the proportion of positive scans either for amyloid or dopamine deficiency was assessed with Fisher’s exact test. Kruskall-Wallis test was used to compare the mean SUVr values across diagnostic groups and for the combined groups (eg. AD and DLB). If the Kruskall-Wallis test detected a significant overall difference, then Wilcoxon rank sum test was applied to compare the following groups: DLB vs. AD, DLB vs. HC, PD vs. AD, PD vs. HC. Linear regression was used to explore the relationship between measures of motor and cognitive performance and imaging data. Receiver operator characteristic (ROC) analysis was used to test the discrimination between groups. The optimal cut-off point was identified using Youden Index method. Fleiss’ kappa was calculated for both imaging modalities to assess inter-rater reliability [23].

## Results

### Demographic features

The study included 11 subjects clinically diagnosed with DLB, 10 with AD, 5 with PD, and 5 HC subjects (Table 1). The mean age did not differ significantly among the 4 groups. The gender distribution varied by group, with a higher proportion of men in the Lewy body disorders group (PD and DLB) compared to AD and HC (p = 0.0558). Over 80% of participants had at least a college education. The combined PD and DLB group demonstrated greater degrees of parkinsonism with higher mean scores on the UPDRS compared to AD and controls (51.81 vs. 13.07; p < 0.0001). The combined AD and DLB group had greater cognitive impairment than the PD and control group (19.19 vs. 29.70, respectively; p < 0.0001). The mean MMSE score for the DLB group was 18.36 (sd 8.94), and the mean MMSE score for the AD group was 20.1 (sd 3.57).

**Table 1:** Demographics characteristics by clinical diagnosis

	**HC**	**PD**	**AD**	**DLB**	**P-value**	Pair-wise comparison*
	**(N = 5)**	**(N = 5)**	**(n = 10)**	**(n = 11)**		
Age	66.40 (11.67)	68.20 (7.76)	75.70 (9.46)	70.18 (9.02)	0.2333	
% female	100	20	50	36.36	0.0558	a,c
Education (number of years. high school = 12, college = 16)	16.40 (0.89)	17.20 (1.10)	15.40 (2.50)	15.09 (3.15)	0.4252	
Disease duration** mean (range)	NA	1.94 (0.93-2.75)	2.08 (0.35-4.47)	1.98 (0.74-3.65)	0.9944	
MMSE; mean (SD)	29.60 (0.55)	29.80 (0.45)	20.10 (3.57)	18.36 (8.94)	0.0002	b,c,d,e
UPDRS; mean (SD)	1.40 (2.61)	30.60 (9.74)	18.90 (20.72)	61.46 (20.54)	<0.0001	a,b,c,d,e,f

MMSE = mini-mental status examination; UPDRS = Unified Parkinson Disease Rating Scalep-value from Kruskal-Wallis test and Fisher exact test.

*Letters indicate pairwise comparisons significant at p < 0.05; a = HC vs. PD; b = HC vs. AD; c = HC vs. DLB; d = PD vs. AD; e = PD vs. DLB; f = AD vs. DLB.

**Years since diagnosis.

### Florbetapir and florbenazine binding

Mean cortical florbetapir SUVr values were highest in the AD group (SUVr =1.53; sd 0.184), followed by the DLB group (1.32; sd 0.285; Table 2). As hypothesized, the mean florbetapir SUVr was higher in the combined AD and DLB group than in the combined PD and HC group (1.42 vs. 1.07; p = 0.0006).

**Table 2:** Florbetapir SUVr for selected cortical regions by diagnostic group

	**HC**	**PD**	**AD**	**DLB**	**P value**	Pair-wise comparison*
Average of cortical regions	1.02 (0.096)	1.12 (0.249)	1.53 (0.184)	1.32 (0.285)	0.0047	b,d
Frontal cortex	0.91 (0.046)	0.98 (0.219)	1.34 (0.204)	1.15 (0.285)	0.0059	b,d
Temporal cortex	1.06 (0.080)	1.11 (0.197)	1.57 (0.191)	1.37 (0.283)	0.0030	b,d
Parietal cortex	0.98 (0.097)	1.01 (0.335)	1.42 (0.188)	1.21 (0.235)	0.0111	b,f
Anterior cingulate	1.00 (0.087)	1.14 (0.241)	1.62 (0.245)	1.32 (0.345)	0.0038	b,d,f
Posterior cingulate	1.03 (0.125)	1.14 (0.312)	1.47 (0.212)	1.38 (0.300)	0.0131	b,c,d
Precuneus	1.16 (0.192)	1.30 (0.302)	1.77 (0.219)	1.51 (0.353)	0.0057	b,d

*a = HC vs. PD; b = HC vs. AD; c = HC vs. DLB; d = PD vs. AD; e = PD vs. DLB; f = AD vs. DLB.

Mean SUVr values (and SD) are given for each group. P-values are for the overall group comparison from Kruskal-Wallis test. Subsequent pair-wise comparisons are noted by the letters in the last column if the overall comparison was significant at p < 0.05.

Mean striatal florbenazine binding was lowest (most abnormal) in the PD group (SUVr = 1.72; sd = 0.172), followed by the DLB group (SUVr = 1.87; sd = 0.372). Mean striatal florbenazine binding in AD subjects was similar to controls (3.04; sd 0.579 and 2.84; sd 0.552, respectively). There were significant group differences in mean striatal SUVr for the combined PD and DLB group compared to the combined AD and control group (1.83 vs. 2.97; p < 0.0001). Among striatal sub-regions, the greatest difference between groups was observed for the lowest posterior putamen (Table 3).

**Table 3:** Florbenazine SUVR by Clinical Diagnosis for each striatal region

	**HC**	**PD**	**AD**	**DLB**	**P value**	**Pair-wise comparison**
Whole striatum*	2.84 (0.552)	1.72 (0.172)	3.04 (0.579)	1.87 (0.372)	0.0001	a, c, d, f
Caudate	2.60(0.529)	1.77 (0.415)	2.21 (0.458)	1.79 (0.418)	0.0303	c, f
Anterior putamen	2.98 (0.601)	1.86 (0.084)	3.39 (0.744)	2.05 (0.519)	0.0003	a, c, d, f
Posterior putamen	2.93 (0.529)	1.53 (0.104)	3.52 (0.758)	1.78 (0.400)	<0.0001	a, c, d, f
Lower posterior putamen**	2.82 (0.474)	1.46 (0.120)	3.41 (0.753)	1.67 (0.427)	<0.0001	a, c, d, f
Laterality index***	0.94 (0.029)	0.91 (0.043)	0.94 (0.032)	0.88 (0.105)	0.5582	
Caudate/putamen ratio****	0.88 (0.035)	1.15 (0.240)	0.64 (0.151)	1.03 (0.240)	0.0011	b, d, f

*Average of left and right caudate, anterior putamen and posterior putamen.

**Posterior putamen with lower SUVr for each subject.

***Ratio of lower to higher posterior putamen for each subject. A higher value indicates greater asymmetry.

****Ratio of average caudate (left and right) to average posterior putamen (left and right). A higher value indicates greater difference between the caudate and posterior putamen.

Mean SUVr values (and SD) are given for each group. P-values are for the overall group comparison from Kruskal-Wallis test. Subsequent pair-wise comparisons are noted by the letters in the last column if the overall comparison was significant (p value < 0.05; a = HC vs. PD; b = HC vs. AD; c = HC vs. DLB; d = PD vs. AD; e = PD vs. DLB; f = AD vs. DLB).

**Figure 1:**
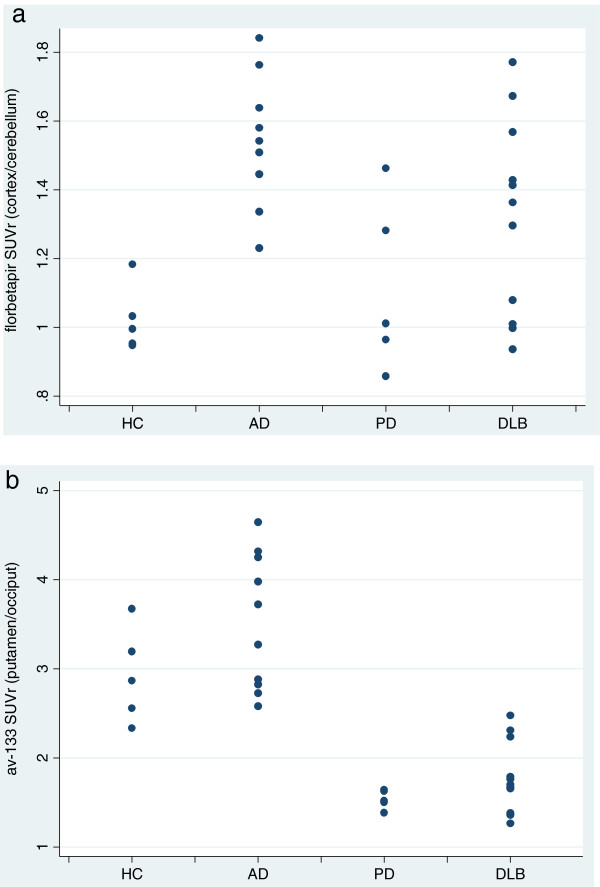
**Scatterplots showing a) average cortical florbetapir SUVr values for each group and b) florbenazine binding for the lowest posterior putamen region for each group.** See text for description of discrimination between groups.

There were some cases of overlap in florbetapir SUVr values between patients in the 4 diagnostic groups (Figure 1a). All AD cases and 7/11 DLB cases had mean cortical florbetapir SUVr values greater than the published cut-off of 1.1 SUVr units [12]. 4/5 controls and 3/5 PD patients were below this cut-off. The area under the ROC curve for florbetapir mean cortical SUVr for the comparison between the combined AD and DLB group and the combined PD and control group was 0.855 (p < 0.0001). There was less overlap for florbenazine between combined PD and DLB subjects compared to combined AD subjects and controls (Figure 1b). For this contrast, ROC analysis using the posterior putamen region showed an AUC of 0.996 (p = <0.0001). The florbenazine SUVr cut-off for the posterior putamen region that provided the best balance of sensitivity and specificity for PD/DLB patients vs. AD patients and HC was 2.3, and resulted in sensitivity of 88% (95% CI: 64%-97%) and specificity of 100% (95% CI: 70%-100%).

### Binary clinical interpretation

The clinical reading for the florbetapir scan was positive in 9 of 10 cases in the AD group and 7/11 cases in the DLB group. It was negative in 4/5 PD patients and all HCs. Overall, the florbetapir qualitative reading was positive in 17/20 cases when the mean cortical SUVr was above 1.1 SUVr units and negative 11/11 cases where the mean cortical SUVr was at or below 1.1 SUVr units, or over 90% correspondence to the published cut-off. For florbenazine, the majority clinical reading was positive for dopamine degeneration in all PD cases and 8/11 DLB case, and negative in all AD cases and HCs. The posterior putamen SUVr cut-off that produced the best agreement with visual interpretation for was 2.12, which is lower than the cut-off that produced the greatest balance of sensitivity and specificity for differentiating between clinical groups (SUVr = 2.3).

Fleiss’ kappa for the binary interpretation among the 5 expert raters was 0.88 (95% CI 0.77-0.99) for florbetapir and 0.95 (95% CI 0.84-1.00) for florbenazine. When the analysis is confined to only demented subjects (AD and DLB, n = 21), Fleiss kappa was 0.90 (95% CI 0.77-1.00) for florbetapir and was 0.92 (95% CI 0.78-1.00) for florbenazine.

### Combined amyloid and dopamine imaging

**Figure 2:**
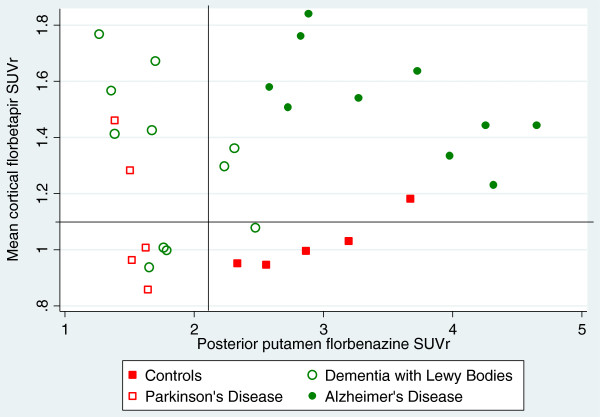
**Pattern of scan results for florbetapir and florbenazine for subjects in the 4 diagnostic groups.** The vertical line shows the florbenazine SUVr cut-off (2.12) that agrees best with expert visual interpretation. The horizontal line (SUVr = 1.1) marks the published quantitative cut-off for florbetapir. The distribution of imaging patterns (proportion of subjects in each quadrant) differed significantly between groups (chi2 = 41.7, p <0.001) In the AD, PD and HC groups, there was one dominant pattern (for example, all of the AD cases had positive florbetapir scans and negative florbenazine scans). While VMAT2 binding was low in the DLB group on average, three subjects had SUVr values above the cut-off, and no single imaging pattern was observed in a majority of DLB patient.

Based on majority binary visual interpretations, four patterns of scan results were possible: AB+/DD+; AB+/DD-; AB-/DD+; AB-/DD-. There were significant differences between groups in the frequency of these patterns (p < 0.001). 9 out of 10 subjects with AD had a florbetapir scan read as positive and a florbenazine scan read as negative , 5/5 controls were negative on both scans. 4/5 PD patients had normal florbetapir imaging and abnormal florbenazine scans. By contrast, the DLB group had heterogeneous imaging results: 5/11 were abnormal on both measures; 3/11 had abnormal florbenazine and normal florbetapir; 2/11 had normal florbenazine and abnormal florbetapir and 1 case was normal on both measures. There were also significant differences between groups in the pattern of amyloid and dopamine abnormalities using quantitative analysis rather visual interpretation (p < 0.001; Figure 2).

### Correlation with clinical variables

We did not find a significant correlation between severity of parkinsonism and florbenazine binding in either the PD or DLB groups (data not shown). Likewise, there was no significant correlation between florbetapir binding and cognition in the PD, AD or HC groups. However, within the DLB group, cases with a positive florbetapir scan based on binary clinical interpretation had significantly lower MMSE scores (14.4 vs. 25.3; p = 0.046), and there was a trend toward a significant correlation between MMSE score and mean cortical florbetapir SUVr in this group (r = −0.54; p = 0.0842). There was a significant association between mean striatal florbenazine binding and MMSE in the DLB group (r = 0.73; p = 0.011). The striatal sub-region that had the strongest association with MMSE was with the caudate (r = 0.72; p = 0.012), while the correlation between MMSE and the posterior putamen was not significant (r = 0.39; p = 0.242). When considering the two biomarkers together, a model containing caudate florbenazine SUVr and mean cortical florbetapir SUVr explained 56% of the variance in MMSE in the DLB group (adjusted R^2^ = 0.559, p = 0.0156, for model fit).

### Safety results

There were no treatment-related adverse events. One subject died 5 days after florbenazine PET. The subject had been in hospice, and the study investigator judged that the death was unlikely to be study-related. In addition, 3 subjects experienced at least 1 treatment emergent AE, none of which were judged to be treatment related. Two subjects in the DLB group had urinary tract infections and one subject in the AD group had a patella fracture related to a fall.

## Discussion

We found significantly greater amyloid deposition in patients with AD or DLB compared to PD patients or controls. We also found significant reductions in VMAT density in PD and DLB patients compared to AD patients or controls. In addition, there was significant inter-rater reliability for both florbetapir and florbenazine.

A characteristic pattern of amyloid and dopamine imaging was present in nearly all subjects with PD and AD. These results are consistent with prior studies in which the frequency of amyloid deposition in PD is similar to age-matched asymptomatic individuals in the general population [24-27]. We found no cases with a clinical diagnosis of AD with abnormal florbenazine imaging. Other studies have reported similarly high specificity (90-97%) for dopaminergic imaging for the detection of DLB vs. AD [28,29].

Our finding of heterogeneous imaging results in DLB patients is also consistent with the existing literature. Another study that found 4/14 cases with clinical DLB had normal dopaminergic imaging, and two of these cases had normal amyloid imaging, as well [30]. In the absence of autopsy confirmation, the discrepancy between clinical diagnosis and imaging cannot be fully resolved. Clinical misdiagnosis may be the most likely explanation. Autopsy studies show that up to 50% of cases clinically diagnosed with DLB in life actually have AD [31]. One imaging study with autopsy follow-up showed that 7/8 cases with a clinical diagnosis of DLB and normal dopaminergic imaging had a diagnosis of AD at autopsy [32]. Nonetheless, the possibility of correctly diagnosed DLB with a normal florbenazine PET scan cannot be entirely excluded. Absence of substantial nigra cell loss is observed in approximately 10% of DLB cases at autopsy [33]. An imaging study with autopsy follow-up found 7 clinical DLB cases with normal dopamine transporter SPECT imaging. Two of these cases had DLB at autopsy (while the remainder had AD). These 2 cases had preserved cell counts in the substantia nigra in spite of the presence of synuclein pathology [34]. Thus, dopaminergic imaging may be normal in DLB cases with synuclein pathology but without extensive nigral degeneration.

Within the DLB group, increasing cognitive dysfunction was associated with decreased VMAT density and greater amyloid deposition. While a link between cognitive dysfunction and dopaminergic denervation has not been consistently reported for DLB, dopaminergic abnormalities have been associated with the presence of neuropsychiatric features in DLB. [35] In addition, an association between dopaminergic imaging abnormalities and worse cognitive performance in PD with dementia (PDD) patients has been reported [36,37]. In longitudinal studies, both amyloid deposition and dopamine deficiency have been associated with the risk of cognitive decline in patients with PD. [38,39] Our study may have been particularly suited to identify an association between cognition and florbenazine imaging because of the large variance in both of these parameters in the DLB patients. For example, MMSE scores in our DLB patients ranged from 4–28. Larger studies with more detailed cognitive testing and longitudinal follow up are needed to confirm our findings. It is possible that dopamine and/or amyloid imaging could identify clinically and biologically relevant subgroups of DLB patients.

There was significant inter-rater reliability for both florbetapir and florbenazine. In each case, the reliability would be considered excellent based on the standards proposed by Landis and Koch although the number of cases interpreted were small [40]. There was also agreement of the florbetapir majority read with the published cut-off for florbetapir [12]. Prior studies [12,22] have demonstrated significant reliability for florbetapir in cohorts consisting of cases of established AD, age-matched controls and young, healthy volunteers. Our results extend these findings to a cohort with a mix of neurodegenerative disorders.

This study needs to be considered in light of several limitations, particularly a small sample size and limited clinical assessments. It is possible that we may have observed more specific effects on cognition if more detailed testing was performed. Second, even though motor testing with the UPDRS was done while patients had not taken their regular medications, the effects of treatment may not have been completely washed out. This factor may partially explain why we did not find a statistically significant relationship between parkinsonism and VMAT density in either the PD or DLB groups. Finally, there is no autopsy confirmation of the diagnosis of our subjects. Autopsy diagnosis is particularly important in DLB where there is substantial clinical, biomarker and pathological heterogeneity.

The results of this study suggest several areas of future investigation. Given the very small number of subjects included per group, these are only preliminary findings. A larger series, possibly with post-mortem diagnostic confirmation, will be required to confirm the findings. Larger studies could further evaluate the potential of combined amyloid and dopamine imaging in differentiating AD and DLB. In the case of DLB, studies in independent cohorts could confirm our finding that the degree of dopaminergic degeneration correlates with greater cognitive impairment. Importantly, the presence of co-existing amyloid may identify a subset of DLB patients with distinct pathology who may have different prognosis or who might be candidates for treatment trials of anti-amyloid therapies.

## Conclusions

The results of this study show that the combination of dopaminergic and amyloid imaging has the potential to identify distinct biomarker signatures among patients with PD, DLB and AD. In particular, there appears to be heterogeneity in the biomarker abnormalities observed in patients with a clinical diagnosis of DLB. These findings may have implications for the prognosis and treatment of patients with these disorders.

## Competing interests

Andrew Siderowf, Michael J. Pontecorvo, Mark A. Mintun, Anupa Arora, Abhinay D. Joshi, Ming Lu and Daniel M. Skovronsky are employed by Avid Radiopharmaceuticals, a subsidiary of Eli Lilly & Co.

Dr. Holly A Shill has research support from Schering-Plough/Merck, Avid Radiopharmaceuticals, UCB Biosciences, Adamas Pharmaceuticals, International Essential Tremor Foundation, Michael J Fox Foundation for Parkinson Research, US Department of Defense and National Institutes of Health.

Charles H. Adler Consultant: Xenoport, Impax, Teva, Ipsen, and Merz. Research support from the Arizona Biomedical Research Commission (contracts 4001, 0011, 05–901 and 1001 to the Arizona Parkinson’s Disease Consortium), the National Institute of Neurological Disorders and Stroke (U24 NS072026 National Brain and Tissue Resource for Parkinson’s Disease and Related Disorders), the Michael J. Fox Foundation for Parkinson’s Research, the Mayo Clinic Foundation, Avid Radiopharmaceuticals, and Phytopharm.

Douglas Galasko Dr. Galasko serves as Editor of Alzheimer^1^s Disease Research and Treatment; serves on Data Safety Monitoring Boards for Elan, Janssen and Balance Pharmaceuticals; is a consultant for Elan Pharmaceuticals. He receives research support from the NIH, the Michael J Fox Foundation and the Alzheimer^1^s Drug Discovery Foundation.

Carolyn Liebsack has nothing to disclose.

Marwan N. Sabbagh Grants/ Contracts: Pfizer, Eisai, Neuronix, Lilly, Avid, Piramal, GE, Avanir, Elan, Functional Neuromodulation, Advisory: Biogen, Lilly, Piramal, Eisai; Royalties: Wiley, Tenspeed (RandomHouse).

## Authors’ contributions

AS have made substantial contributions to conception and design, or acquisition of data, or analysis and interpretation of data; have been involved in drafting the manuscript or revising it critically for important intellectual content; MJP have given final approval of the version to be published. HAS have made substantial contributions to conception and design, or acquisition of data, or analysis and interpretation of data; have been involved in drafting the manuscript or revising it critically for important intellectual content; MAM have given final approval of the version to be published. AA have made substantial contributions to conception and design, or acquisition of data, or analysis and interpretation of data; ADJ have made substantial contributions to conception and design, or acquisition of data, or analysis and interpretation of data; ML have made substantial contributions to conception and design, or acquisition of data, or analysis and interpretation of data; CHA have made substantial contributions to conception and design, or acquisition of data, or analysis and interpretation of data; DG have been involved in drafting the manuscript or revising it critically for important intellectual content; CL have made substantial contributions to conception and design, or acquisition of data, or analysis and interpretation of data; DMS have given final approval of the version to be published. MNS have made substantial contributions to conception and design, or acquisition of data, or analysis and interpretation of data; have been involved in drafting the manuscript or revising it critically for important intellectual content. All authors read and approved the final manuscript.

## Pre-publication history

The pre-publication history for this paper can be accessed here:

http://www.biomedcentral.com/1471-2377/14/79/prepub
